# Uncovering dual molecular diagnoses in families with complex phenotypes through structural and clinical studies of novel *COL4A6* variants

**DOI:** 10.1093/qjmed/hcaf246

**Published:** 2025-10-15

**Authors:** Daniel Owrang, Aboulfazl Rad, Constantin Cretu, Sheng-Jia Lin, Hafiz Muhammad Mustafa, Kevin Huang, Nadia Waheed, Maqbool Hussain, Sadia Riaz, Julia Preobraschenski, Gaurav K Varshney, Gabriela Oprea, Barbara Vona

**Affiliations:** Institute for Auditory Neuroscience and InnerEarLab, University Medical Center Göttingen, Göttingen, Germany; German Primate Center, Auditory Neuroscience and Optogenetics Laboratory, Göttingen, Germany; Collaborative Research Center 1690 (CRC1690), University of Göttingen, Göttingen, Germany; Institute of Human Genetics, University Medical Center Göttingen, Göttingen, Germany; Arcensus GmbH, Rostock, Germany; Institute for Auditory Neuroscience and InnerEarLab, University Medical Center Göttingen, Göttingen, Germany; Cluster of Excellence “Multiscale Bioimaging: from Molecular Machines to Networks of Excitable Cells” (MBExC), University of Göttingen, Göttingen, Germany; Genes and Human Disease Research Program, Oklahoma Medical Research Foundation, Oklahoma City, OK, USA; University of Karachi, Pakistan; Genes and Human Disease Research Program, Oklahoma Medical Research Foundation, Oklahoma City, OK, USA; Children Hospital, Pakistan Institute of Medical Sciences, Islamabad, Pakistan; Children Hospital, Pakistan Institute of Medical Sciences, Islamabad, Pakistan; Children Hospital, Pakistan Institute of Medical Sciences, Islamabad, Pakistan; Institute for Auditory Neuroscience and InnerEarLab, University Medical Center Göttingen, Göttingen, Germany; Collaborative Research Center 1690 (CRC1690), University of Göttingen, Göttingen, Germany; Cluster of Excellence “Multiscale Bioimaging: from Molecular Machines to Networks of Excitable Cells” (MBExC), University of Göttingen, Göttingen, Germany; Genes and Human Disease Research Program, Oklahoma Medical Research Foundation, Oklahoma City, OK, USA; Arcensus GmbH, Rostock, Germany; Institute for Auditory Neuroscience and InnerEarLab, University Medical Center Göttingen, Göttingen, Germany; German Primate Center, Auditory Neuroscience and Optogenetics Laboratory, Göttingen, Germany; Collaborative Research Center 1690 (CRC1690), University of Göttingen, Göttingen, Germany; Institute of Human Genetics, University Medical Center Göttingen, Göttingen, Germany; Department of Obstetrics and Gynecology, Brigham and Women’s Hospital, Harvard Medical School, Boston, MA, USA; Program in Medical and Population Genetics, Broad Institute of MIT and Harvard, Cambridge, MA, USA

## Abstract

**Background:**

The relationship between observed clinical phenotypes and underlying genotypes is blended or skewed in multiple molecular diagnoses, complicating a comprehensive molecular genetic diagnosis.

**Aim:**

We report two families with dual diagnoses, using the deafness-associated gene, *COL4A6*, to exemplify its contribution to blended, complex clinical presentations.

**Design:**

This is an observational study within a large, ethnically diverse rare disease cohort, focusing on families with hearing loss and suspected dual diagnoses, followed by functional and structural studies of novel variants.

**Methods:**

Families were identified through a large rare disease sequencing initiative. Exome or genome sequencing was performed, with follow-up RNA studies for a synonymous *COL4A6* variant. Spatial and temporal expression analysis in zebrafish traced *col4a6* expression in the otic vesicle and ear from 1 to 5 days post-fertilization. Structural modeling was used to estimate variant impact on protein structure.

**Results:**

We identified two families affected by multiple genetic disorders. The first family presented a missense *COL4A6* variant (NM_033641.4: c.1480G>A p.(Gly494Arg)), accounting for hearing loss, while a likely pathogenic *HEXA* variant (NM_000520.6: c.902T>G p.(Met301Arg)) explained Tay-Sachs disease features. The second family exhibited a synonymous *COL4A6* variant (NM_033641.4: c.1767G>A p.(Pro589=)), leading to partial exon skipping and hearing loss, along with a pathogenic splice-site variant in *DYM* (NM_001353214.3: c.1125 + 1G>T p.?), causing the Dyggve-Melchior-Clausen disease.

**Conclusions:**

Our findings highlight the importance of recognizing dual molecular diagnoses to untangle blended phenotypes, as well as the diagnostic relevance of synonymous variants with predicted splicing effects.

## Introduction

Hereditary hearing loss (HL) is a prevalent condition with significant genetic and clinical heterogeneity,^1^ and more than 150 genes are presently implicated in non-syndromic HL. X-linked NSHL is particularly rare, affecting ∼1–5% of HL patients, and only five X-linked genes (*PRPS1*, *POU3F4*, *SMPX*, *AIFM1*, and *COL4A6*) have been associated with auditory function.^2^^,^^3^ Among these, *COL4A6* (MIM #303631) is causal for DFNX6 (MIM #300914), a form of X-linked non-syndromic HL initially identified in a Hungarian family exhibiting severe prelingual bilateral sensorineural HL with cochlear malformation in males and late-onset, mild to moderate HL in females.^2^

*COL4A6* encodes a critical component of type IV collagen, called collagen type IV alpha-6, and forms basement membranes in multiple tissues.^3^ Immunohistochemical staining of the mouse inner ear at 12 weeks of age showed *Col4a6* expression in the membranous and osseous structures of the stria vascularis of the spiral ligament and in a subgroup of the spiral ganglia cells. Additionally, whole-mount *in situ* hybridization (WISH) of *col4a6* in zebrafish embryos of various stages revealed *col4a6* expression in the nervous system and auditory structures, notably in the otic vesicle, swim bladder and lateral line.^2^ Collagen IV, like all collagen superfamily members, displays the characteristic triple helical structure of three alpha chains that are tightly wound around each other. Collagen IV forms hexameric structures composed of two *COL4A6* and *COL4A5* heterotetramers.^2^ Glycine residues in Gly-X-Y repeats stabilize the structure through interchain hydrogen bonds and hydrophobic and electrostatic interactions.^4^

So far, reported variants in patients involve disruption to Gly-X-Y motifs. In a gene discovery study involving a Hungarian family, protein stability and structure prediction revealed a striking destabilization of the collagen triple-helix due to the p.Gly591Ser variant. Another study reported two additional families with males diagnosed with X-linked HL.^2^ One had a rare hemizygous missense *COL4A6* variant (NM_001287758.1: c.3272G>C (p.Gly1091Ala)). In the other, aberrant splicing caused by the c.951 + 1G>T variant led to loss of exon 15 and a 15-amino acid deletion of Gly-X-Y residues.^5^

The large number of genes involved in normal hearing make HL a frequent feature in cases of multiple molecular diagnoses, when more than one genetic aberration explains the complete clinical constellation of a patient, or dual diagnoses, when two distinct loci are involved.^6–8^ Such cases have been reported in several studies, with an estimated frequency of ∼5–7% of diagnoses.^9^^,^^10^ Considering dual diagnoses are essential in complex or atypical presentations, not only to support accurate variant interpretation, but also to ensure comprehensive clinical diagnoses.^11^ These insights are critical for guiding appropriate management and realizing the goals of genomic medicine, namely, particularly precision therapies tailored to an individual’s full genetic findings.^9^

This study presents two families with dual molecular diagnoses identified within a large, ethnically diverse rare disease cohort, with a focus on families affected by HL. Functional and structural studies were performed to evaluate the impact of novel variants.

## Materials and Methods

### Recruitment and clinical assessment

This study was approved by the Ethics Committee of Rostock University, A2022-0072, 25.04 2022 and by the Ethical Review Board of Pakistan Institute of Medical Sciences. Written informed consent was obtained from all participants or from parents of minors.

The probands in families 1 and 2, both from consanguineous Pakistani parents, were identified during routine diagnostics at Arcensus GmbH and considered as part of a collaborative effort supporting a large ethnically diverse rare disease study. Inclusion criteria required a dual molecular diagnosis involving *COL4A6* and an additional locus.

Proband clinical data, including demographic, otolaryngologic, audiological, and medical records, were reviewed. In family 2, individuals III:2, IV:1, and IV:4 underwent pure-tone audiometry, measuring hearing thresholds at 0.25, 0.5, 1, 2, 4, 6, and 8 kHz. HL severity was classified by calculating pure tone averages (PTA) of frequencies between 0.5 and 4 kHz (PTA_0.5-4k_) to determine the severity of HL as mild (21–40 dB), moderate (41–70 dB), severe (71–95 dB), or profound (>95 dB).^12^ Unilateral or asymmetric HL is defined as a >10 dB difference between the ears in at least two frequencies over 0.5, 1 and 2 kHz. Brain MRI was performed for individual IV:4 in family 2.

### Genome/exome sequencing, variant interpretation, and validation

Genomic DNA was isolated from whole blood using standard procedures from family 1 (II:3, II:4, II:5, III:1) and family 2 (III:1, III:2, IV:1, IV:4).

Exome sequencing for proband III:1 (family 1) was performed using TruSeq Rapid Exome library preparation (Illumina, San Diego, CA, USA) and sequenced on a NextSeq 500 (Illumina). De-multiplexed data were mapped to GRCh38/hg38 using BWA. Proband IV:4 (family 2) underwent genome sequencing using TruSeqNano DNA High Throughput Library preparation (Illumina) and sequenced using pair-end 150 bp reads on an Illumina platform, yielding an average 30× nuclear and ≥1000× mitochondrial genome coverage. Reads were aligned to GRCh38/hg38 and variant calling with default parameters were performed using DRAGEN (version 3.10.4, Illumina). Single nucleotide substitutions and small insertions/deletions annotation were performed by standard variant interpretation tools. Structural variants were annotated with ANNOTSV3.1 and an in-house structural variant database to obtain allele frequencies. Mitochondrial variants with frequencies ≥5% were detected. Variant descriptions follow the Human Genome Variation Society recommendations (www.hgvs.org).

Variants were filtered using allele frequencies from the Exome Variant Server, gnomAD v4.1.0, and dbSNP, with thresholds above 0.005 (recessive) and 0.0005 (dominant or X-linked HL-associated genes). Interpretation included Combined Annotation Dependent Depletion (CADD) v1.7, MutationTaster, REVEL, SIFT v6.2.1, and PolyPhen-2 v2.2.3. Public databases used for pathogenicity assessment included ClinVar, the Human Gene Mutation Database (HGMD), the Deafness Variation Database v9, All of Us, and deCAF. Splicing predictions used SpliceSiteFinder-like, MaxEntScan, NNSPLICE, and GeneSplicer through AlamutVisualPlus v1.12 with a ≥10% reduction in splice site strength compared to reference considered supporting evidence of splice disruption. For SpliceAI/Pangolin and Absplice, this corresponded to scores ≥0.20 and ≥0.01, respectively. All *in silico* predictions were interpreted in the context of gene structure and known disease mechanisms. Variant classifications followed the American College of Medical Genetics and Genomics/Association for Molecular Pathology (ACMG/AMP) guidelines,^13^ and were tailored to the ClinGen HL expert specifications, as applicable.^14^

Segregation analysis was performed by Sanger sequencing with standard PCR sequencing parameters. Primers are listed in Supplementary Table 1.

### Structural modeling of a *COL4A6* variant

To assess the potential structural impact of the Gly494Arg substitution, an idealized model of the collagen IV triple-helix was constructed using the BuScr script (v1.07a).^15^ The model assumed a heterotrimeric configuration composed of two α5(IV) chains and one α6(IV) chain, consistent with the known structure of type IV collagen.

The amino acid side chains, including the Gly494Arg substitution, were manually incorporated using ChimeraX v1.8^16^ with the Dunbrack rotamer library,^17^ followed by correction for steric clashes.^18^ Local melting temperatures of the modeled residues were estimated using the BuScr to evaluate potential destabilization effects. Structural visualization and figure generation were performed in ChimeraX.

This approach focused on a representative fragment surrounding the variant site, capturing the characteristic Gly-X-Y motif of the collagenous domain to ensure a structurally relevant context for assessing local disruptions.

### Minigene assay

RNA studies were performed as previously described with modifications.^19^^,^^20^ A 645 bp genomic region of *COL4A6* containing 107 bp from intron 21, exon 22 (180 bp), and 358 bp from intron 22 was amplified from the proband (family 2, IV:4) and control DNA using restriction-site-containing primers (Supplementary Table 1). The PCR fragment was ligated between exons A and B of the linearized pSPL3 vector following digestion with restriction enzymes. The recombinant vectors were transformed into DH5α competent cells (NEB 5-alpha, New England Biolabs, Frankfurt, Germany), plated, and incubated overnight. Following colony PCR with SD6 F and a *COL4A6*-specific reverse primer, the wild-type and mutant-containing vector sequences were confirmed by Sanger sequencing and transfected into HEK 293 T cells (ATCC, Manassas, VA, USA) at a density of 2 × 10^5^ cells per mL; 2 µg of the respective pSPL3 vectors was transiently transfected using 6 µL of FuGENE 6 Transfection Reagent (Promega, Walldorf, Germany). Empty vector and transfection negative reactions were included as controls. Cells were harvested 24 h after transfection. Total RNA was prepared using the miRNeasy Mini Kit (Qiagen, Hilden, Germany). RNA was reverse transcribed using the High Capacity cDNA Reverse Transcription Kit (Applied Biosystems, Waltham, MA, USA) following the manufacturer’s protocols. The cDNA was PCR amplified using vector-specific SD6 F and SA2 R primers. The amplified fragments were visualized on a 1% agarose gel and Sanger sequenced.

### Zebrafish expression studies

Zebrafish experiments were conducted in the NHGRI-1 wild-type strain in accordance with the Institutional Animal Care and Use Committee (IACUC) of the Oklahoma Medical Research Foundation^18–22^ approved protocol. All animals were reared and kept under standard conditions in an Association for Assessment and Accreditation of Laboratory Animal Care (AAALAC) accredited facility.

### Wish

WISH was performed using a previously described technique.^21^ In brief, we PCR amplified an 858 bp *col4a6* fragment from zebrafish cDNA using primers that included T3 promoter sequence at the 5′ end of the *col4a6*-specific forward primer and T7 promoter sequence at the 5′ end of the *col4a6* reverse primer (Supplementary Table S1). We utilized the amplified DNA fragments to synthesize the antisense riboprobe labeled with digoxigenin-UTP. We treated wild-type embryos at the 24 hpf stage to prevent pigmentation with 0.003% 1-Phenyl-2-thiourea (Millipore Sigma, MO, USA) and allowed them to develop until the desired developmental stages. The embryos were fixed with 4% (V/V) paraformaldehyde and dehydrated through increasing methanol concentrations before being stored in 100% methanol at −20°C overnight. The next day, we rehydrated the embryos using decreasing methanol concentrations and permeabilized them with 10 µg/ml proteinase K. For color development, we used BM purple alkaline phosphatase substrate (Millipore Sigma, MO, USA).

### RNA extraction and RT-qPCR

Total RNA was extracted from different stages of embryos using the TRIzol Reagent (Thermo Fischer Scientific, Waltham, MA, USA) and purified by the miRNeasy Mini kit (Qiagen, Hilden, Germany), following the manufacturer’s instructions.

The mRNA was reverse-transcribed into cDNA and used as a template for quantitative real time PCR (qRT-PCR) using SYBR Green Supermix as described earlier.^22^ All relative quantifications were done using three independent experimental replicates and three technical replicates per amplification; the values were normalized to the housekeeping gene 18S. The primer sequences for RT-qPCR are shown in Supplementary Table S1.

## Results

### Clinical evaluation

**Figure 1.**
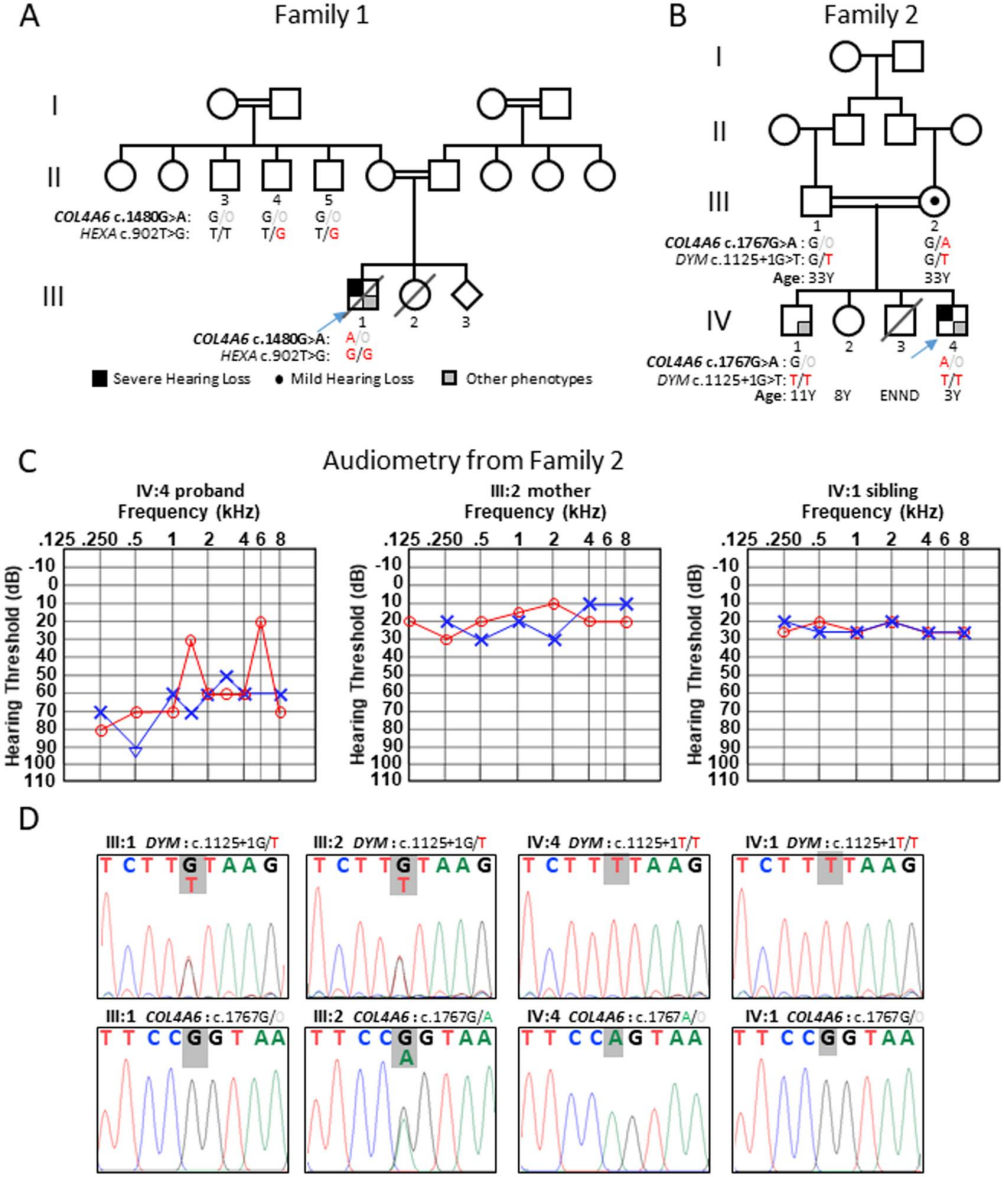
Audiometric data and genetic diagnoses. (**A**) Pedigree of family 1 and (**B**) pedigree of family 2 with symbols representing various clinical features below. The proband of each family is marked with an arrow, and the genetic variants (in red) identified with their segregation are shown. (**C**) Audiogram of three members of family 2. (**D**) Segregation of the homozygous *DYM* (c.1125 + 1G>T) and hemizygous *COL4A6* (c.1767G>A) variants in family 2. Sequences are shown for the proband (IV:4), parents (III:1 and III:2), and sibling (IV:1). Abbreviation: ENND, early neonatal period death (septicemia).

The proband in family 1 (Figure 1A, III:1) was identified through routine clinical evaluations and exome sequencing analysis. Family history of hereditary disorders was negative and the parents were both clinically normal hearing. The 2-year-old proband presented with a cherry-red spot of the macula, developmental regression, microcephaly, feeding difficulties, constipation, fever, recurrent infections, recurrent upper respiratory tract infections, seizures, and vision and hearing impairment. An updated auditory brainstem response test was planned but the proband passed away before testing could be completed. He exhibited normal development without any abnormality until the age of 7 months, after which he began to experience a gradual loss of hearing. By 1.6 years of age, he was completely deaf and had also lost his vision. He had an exaggerated startle response. His complete blood panel, liver functions tests, renal functions tests and serum electrolytes were all within normal range. His bone marrow biopsy was negative for any storage cells. He and his otherwise healthy sister passed away at the age of 6 months and 2 years, respectively due to lower respiratory infection, which is a common cause of infant mortality in Pakistan.

The proband in family 2 (Figure 1B, IV:4) was referred for molecular genetic testing at age 3 years for motor and speech delay, HL, and skeletal anomalies suggestive of mucopolysaccharidosis. He was born after an uncomplicated delivery. Family history was negative in generations I to III (Figure 1B). His mother noticed that he was unresponsive to vocal sounds and later clinically diagnosed with HL at age 3 years with thresholds between 30 and 80 dB. PTA_0.5-4k_ indicated bilateral moderate HL with 65 dB (left) and 57.5 dB (right). Motor milestones were delayed. He began neck control at 6 months of age, sat at 12 months, and walked at 22 months. The mother (III:2) also showed mild HL (borderline normal hearing) only in the left ear at 27 years old with PTA_0.5-4k_ 22.5 dB, while not meeting the definition of asymmetric or unilateral HL, and a brother (IV:1) had normal hearing (Figure 1C). The mother has no speech issues. Unfortunately, the father was unavailable for screening but is clinically healthy and has a full-time job. The proband’s vision was normal. His speech development was severely impacted to the extent that he was nonverbal. He was referred to a tertiary care hospital for detailed assessments. At the age of 3 years, anthropometry showed height 80 cm (<3rd centile), weight 10 kg (<5th centile), and fronto-occipital circumference 48 cm (2 SD). Skeletal findings included short trunk short stature with disproportionate long limbs, short broad thumbs, short neck, and coarse facial features. Biochemical laboratory tests revealed normal liver and renal function, serum electrolytes, vitamin D levels, and urinary glycosaminoglycan levels within normal range. Brain MRI showed normal cortex and white matter. His skull was slightly elongated likely due to premature closure of sagittal suture and a skeletal survey revealed generalized platyspondyly of the spine with a characteristic cervical double hump and anterior vertebral wedging. His older brother had similar skeletal features and motor delay but normal hearing and speech development. One male sibling died in the early neonatal period; no clinical evaluation for the neurodevelopmental or auditory phenotype was possible. He died of septicemia in the early neonatal period.

### Molecular genetic testing

#### Family 1

Exome sequencing revealed a hemizygous missense *COL4A6* variant (NM_033641.4: c.1480G>A p.(Gly494Arg)) (Figure 1A) absent from HGMD or ClinVar and the Deafness Variation Database. Pathogenicity prediction tools scored the variant as follows: CADD: 23.4, REVEL: 0.945, MutationTaster: benign, PolyPhen-2: probably damaging, and SIFT: deleterious (Table 1), with no predicted consequence on splicing. This missense variant affects a moderately conserved nucleotide and amino acid (Table 1). Three maternal uncles have the wild-type *COL4A6* allele (Figure 1A). This variant is classified as likely pathogenic based on ACMG/AMP guidelines (PM1_Moderate, PM2_Supporting, PP3_Strong).

**Table 1:** Details of the variants identified in the probands

	Family 1 proband (III:1)		Family 2 proband (IV:4)	
Reported genetic ancestry	Pakistani		Pakistani	
Sequencing method	Exome		Genome	
Variant details				
	*COL4A6* variant details	*HEXA* variant details	*COL4A6* variant details	*DYM* variant details
Exon number	21 of 45	8 of 14	22 of 45	intron 10 of 17
gDNA Level: (GRCh38)	chrX: g.108188624C>T	chr15: g.72349163A>C	chrX: g.108187848C>T	chr18: g.49281996C>T
gDNA Level: (GRCh37)	chrX: g.107431854C>T	chr15: g.72641504A>C	chrX: g.107431078C>T	chr18: g.46808366C>T
cDNA Level:	NM_033641.4: c.1480G>A	NM_000520.6: c.902T>G	NM_033641.4: c.1767G>A	NM_001353214.3: c.1125 + 1G>A
Protein Level	p.(Gly494Arg)	p.(Met301Arg)	p.(Pro589=)	p.?
Domain	Collagen triple helix repeat	Glycoside hydrolase family 20, catalytic domain	Collagen triple helix repeat	Extracellular domain
Conservation, consequence				
phyloP: [−19.0, 11.0]	6.01 (Moderately conserved)	9.22 (Moderately conserved)	5.66 (Moderately conserved)	7.72 (Highly conserved)
Coding effect	Missense	Missense	Synonymous	Intronic (no effect)
Codon change	GGG to AGG	ATG to AGG	CCG to CCA	not applicable
Variant type	SNV	SNV	SNV	Splicing variant
Consequence	missense variant	missense variant	synonymous with splice_region_variant	splice_region_variant
ACMG/AMP pathogenicity classification	Likely Pathogenic, PM1_Moderate, PM2_Supporting, PP3_Strong	Likely Pathogenic, PM2_Supporting, PP3_Strong, PP2_Supporting	VUS, PM2, BP4	Pathogenic, PVS1_Very strong, PM2_Supporting, PP5_Supporting
Population databases				
(Highest sub-population name-allele frequency) [allele count, number of homozygotes, hemizygotes] (gnomAD v4.1.0)	(East Asian 0.00009) [5,0,3]	(South Asian 0.00018) [19,0,0]	(Middle Eastern 0.0002331) [16,0,5]	(European (Non-Finnish) 8.480e-7) [1, 0]
Number of hemizygotes/allele count (gnomAD v2.1.0)	Not reported	(South Asian 0.00013) [4,0,0]	(South Asian 0.00009) [2,1,0]	Not reported
deCAF (allele count, allele frequency)	British/Irish (1, 3.89e-6)	South Asian (3, 4.92e-4)	British/Irish (2, 7.92e-6)	Not reported
All of us (allele count, allele frequency, homozygote count)	1, 0.000008, 0	Not reported	8, 0.000012, 0	Not reported
Reference databases				
dbSNP	rs1341885508	rs121907977	rs781038243	rs2094993424
ClinVar	Not reported	Likely pathogenic/pathogenic	Not reported	Likely pathogenic
Deafness Variation Database v9	Not reported	Not reported	Unknown significance	Not reported
Splice prediction tools				
SpliceSiteFinder-like	0.0%	0.0%	−13.3%	−100.0%
MaxEntScan	0.0%	0.0%	−16.7%	−100.0%
NNSPLICE	0.0%	0.0%	−3.3%	−100.0%
GeneSplicer	0.0%	0.0%	−54.2%	−100.0%
SpliceAI 10k [≥0.2|0.5|0.8]	0.00	0.00	DG 0.12 (17) DL 0.04	DL 0.99 (1)
AbSplice [≥0.01|0.05|0.2]	< 0.01	< 0.01	0.047 (Artery Tibial)	0.38 (Nerve Tibial)
Pangolin [≥0.2|0.5|0.8]	−0.0300	0.01	0.160	−0.860
Pathogenicity prediction tools				
CADD_Phred	23.4	27.5	12.28	33
REVEL	0.945	0.958	Not available	Not available
MutationTaster	Benign	Deleterious	Not available	Not available
PolyPhen-2	Probably damaging	Probably damaging	Not available	Not available
SIFT	Deleterious	Deleterious	Not available	Not available
Interpretation of variant pathogenicity				
ACMG/AMP Pathogenicity Classification	Likely Pathogenic, PM1_Moderate, PM2_Supporting, PP3_Strong	Likely Pathogenic, PM2_Supporting, PM3_Moderate, PP3_Strong, PP2_Supporting, PP4_Supporting	Likely Pathogenic, PM1_Moderate, PM2_Supporting, PM4_Moderate, PP3_Supporting, PS3_Supporting	Likely Pathogenic, PVS1_Very strong, PM2_Supporting

A likely pathogenic homozygous *HEXA* (MIM: #606869) variant was also identified (NM_000520.6: c.902T>G p.(Met301Arg)) that is associated with Tay-Sachs disease. ClinVar lists conflicting classifications of pathogenicity (Pathogenic [2]; Likely pathogenic [2]; Uncertain significance [1]) and the variant is published as pathogenic in two families. There was no predicted consequence on splicing. Pathogenicity prediction tools scored the variant as follows: CADD: 27.5, REVEL: 0.958, MutationTaster: deleterious, PolyPhen-2: probably damaging, and SIFT: deleterious. This variant is classified as likely pathogenic based on ACMG/AMP guidelines (PM2_Supporting, PM3_Moderate, PP3_Strong, PP2_Supporting, PP4_Supporting) (Table 1).

#### Family 2

Genome sequencing uncovered a novel hemizygous synonymous *COL4A6* variant (NM_033641.4: c.1767G>A p.(Pro589=)). *In silico* splice prediction tools showed mixed predictions which prompted splicing analysis (Table 1): SpliceSiteFinder-like, MaxEntScan, NNSPLICE, and GeneSplicer showed an average of 11% reduction in the native splice donor score, AbSplice predicted a splice donor loss with a score of 0.047, while SpliceAI Visual did not reach significance. Pangolin was just below the threshold, and the CADD score was relatively low at 12.28.

A homozygous likely pathogenic canonical splice-site variant in *DYM* (MIM: #607461) (NM_001353214.3: c.1125+1G>T) (PVS1_VS, PM2_Supporting), was also identified and previously reported to cause Dyggve-Melchior-Clausen disease (DMC). This variant is predicted to abolish normal splicing, with strong support from SpliceAI (score: DL 0.99^1^) and Pangolin (score: −0.860) as well as SpliceSiteFinder-like, MaxEntScan, NNSPLICE, and GeneSplicer (score: −100%), and a high CADD score of 33, consistent with its known pathogenicity (Table 1). The unaffected mother was heterozygous for both variants (Figure 1D). A sibling (IV:1) with normal hearing (Figure 1C) was homozygous for the *DYM* variant and showed features consistent with DMC disease, while the proband exhibited both DMC and HL. The unaffected sister was unavailable for genetic testing.

### Protein modeling

**Figure 2.**
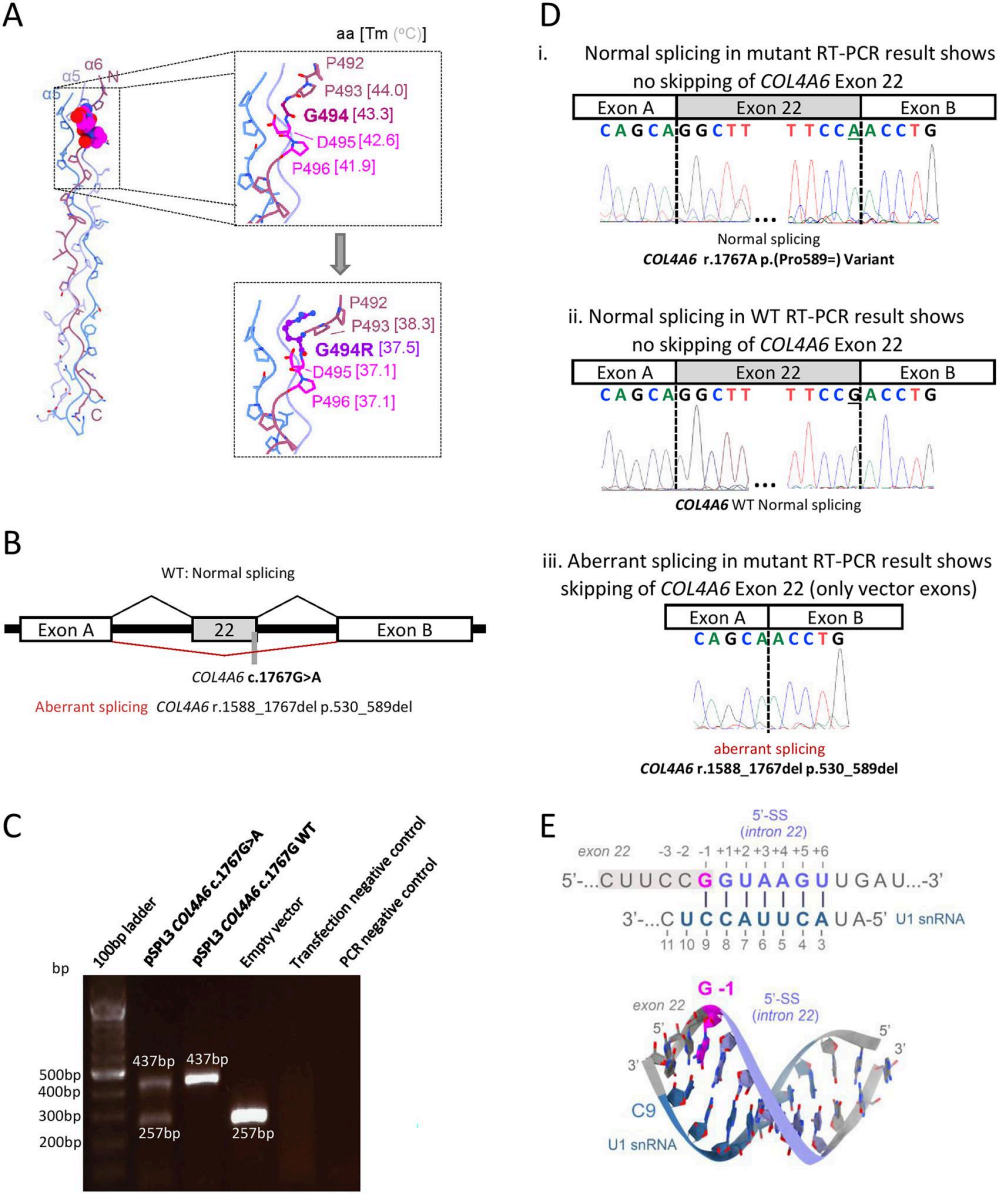
Variant-specific characterization: protein structure modeling and minigene assay of the *COL4A6* c.1767G>A variant. (**A**) Structural consequences of the Gly494Arg substitution. (**B**) The *COL4A6* exon 22 trapping vector construct for the *in vitro* splice assay of c.1767G>A variant with a schematic summary of results. An amplicon containing either mutated or wild-type (WT) exon 22 was inserted between exons A and B in the pSPL3 vector. The black lines above the figure show the wild-type results, while the red lines below summarize the aberrant splicing results. (**C**) Gel electrophoresis of cDNA amplicons from HEK293T cells transfected with the c.1767G>A variant-containing vector, WT, or empty vector. The WT and mutant cDNA amplicons revealed correctly spliced 437 bp amplicons (with less intensity in mutant sample). The mutant also yielded a 257 bp amplicon, identical to the empty vector. Controls performed as expected. (**D**) A schematic of the resulting splice product sequences. The WT and mutant amplicons produce a 437 bp normally spliced product (i, ii) variant position highlighted in the yellow box. The mutant amplicon additionally shows the skipping of exon 22 (iii), indicated by the sequence of the vector exons A and B. (**E**) Proposed mechanism of the aberrant splicing caused by the c.1767G>A variant. Our modeling indicates that the substituted nucleobase (magenta, G-1) maps to the exon 22-intron 22 junction of *COL4A6* and base pairs with C9 of the U1 snRNA, thereby contributing to the formation of the U1-5′ splice site (SS) duplex. The structural model of the U1-5′ SS duplex (bottom panel), formed at the exon 22-intron 22 junction of *COL4A6*, is shown in stick representation. The base-paired pre-mRNA and U1 nucleobases are colored light blue and teal, respectively. Abbreviation: WT, wild-type.

Modeling of the Gly494Arg substitution revealed that replacing the invariant glycine residue within the Gly-X-Y repeat significantly perturbs the collagen IV triple-helix. The introduction of the bulky, positively charged arginine side chain is predicted to induce a local kink and destabilize the helical structure at the variant site. Moreover, local melting temperature estimates indicated a marked reduction in thermal stability around the substitution. Given the critical role of glycine in maintaining the tight packing of collagen helices,^23^ these findings suggest that the Gly494Arg change may compromise the mechanical integrity of collagen IV fibers (Figure 2A).

### Functional validation of the *COL4A6* c.1767G>A p.(Pro589=) variant

*In silico* splicing prediction (Supplementary Table 2) suggested potential aberrant splicing that was tested using an *in vitro* splice assay (Figure 2B and C). This assay confirmed leaky splicing that involved a mixture of normal splicing and exon skipping (r.1588_1767del p. Gly530_Pro589del) causing an in-frame deletion of exon 22 (Figure 2D) in a highly conserved Gly-X-Y repeat region. With added functional studies, this variant is classified as likely pathogenic (PM1_Moderate, PM2_Supporting, PM4_Moderate, PP3_Supporting, PS3_Supporting).

### *col4a6* mRNA expression during zebrafish development

**Figure 3.**
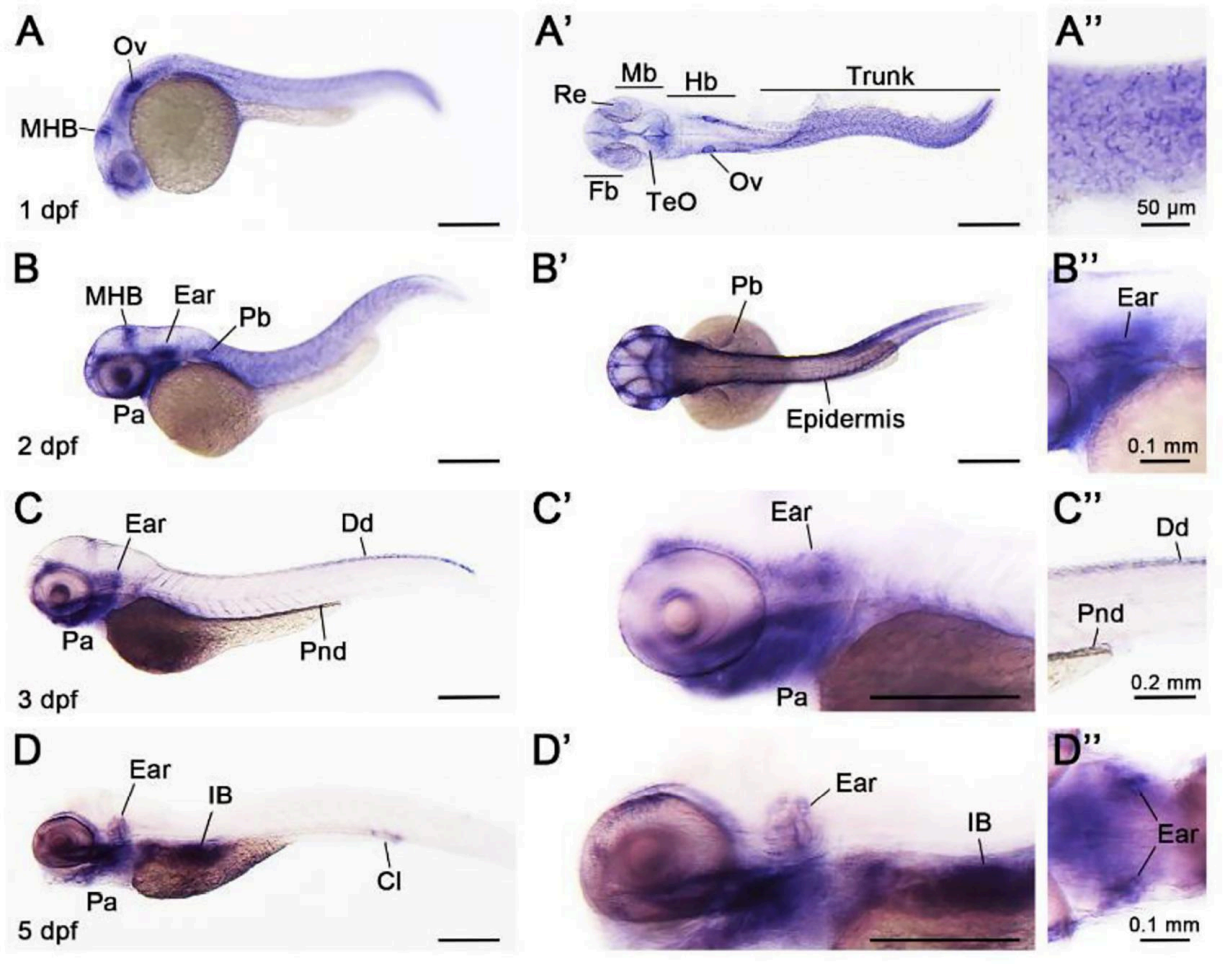
Spatial and temporal expression pattern of *col4a6* mRNA during zebrafish development. (**A**) At 1 day post-fertilization (dpf), *col4a6* mRNA is expressed in the epidermis, otic vesicles (Ov), and midbrain-hindbrain boundary (MHB), where the epidermis is enlarged for clarity as shown in A″. Panel A′ shows a dorsal view of panel A with a flattened mount. (**B**) At 2 dpf, *col4a6* mRNA is persistently expressed in the MHB, ear (enlarged in B″), and epidermis, and is beginning to be expressed in the pharyngeal arch (Pa) and pectoral fin bud (Pb). (**C**) At 3 dpf, *col4a6* mRNA is persistently expressed in the pharyngeal arch and ear (enlarged in C′) and becomes restricted in the dorsal dermis (Dd, enlarged in C″). In addition, *col4a6* mRNA is expressed in the pronephric duct (Pnd). (**D**) At 5 dpf, *col4a6* mRNA is persistently expressed in the pharyngeal arch and ear (enlarged in D′ and dorsal view in D″), with additional expression in the intestinal bulb (IB) and cloaca (Cl). Unlabeled scale bars are 0.3 mm.

We aimed to examine the expression pattern of *col4a6* mRNA during zebrafish development to gain a better understanding of the function of *col4a6*. At 1-day post-fertilization (dpf), *col4a6* mRNA was observed in the epidermis, otic vesicles, and midbrain-hindbrain boundary (Figure 3A-A'). Epidermal expression is also shown in an enlarged image, Figure 3A''. At 2 dpf, ubiquitous expression of *col4a6* mRNA was observed in various tissues, including the midbrain-hindbrain boundary, ear, epidermis, pharyngeal arch, and pectoral fin bud (Figure 3B-B''). By 3 dpf, the mRNA was detected in the pharyngeal arch, inner ear, dorsal dermis, and pronephric duct. Finally, at 5 dpf, *col4a6* mRNA continued to be expressed in the pharyngeal arch, inner ear, intestinal bulb, and cloaca (Figure 3C-C''). Overall, the *col4a6* mRNA is expressed in the inner ear from 1dpf to 5dpf in developing zebrafish. Furthermore, qRT-PCR analysis revealed dynamic expression of *col4a6* during zebrafish development. *col4a6* transcripts were detected as early as 24 h post-fertilization (hpf) and showed stage-dependent changes in expression levels through subsequent stages (Supplementary Figure 1). Expression remains the same at 48 hpf of development, peaking at 72 hpf, followed by a modest decline by 120 hpf. These findings suggest that *col4a6* is developmentally regulated and may play a crucial role in organogenesis during zebrafish development.

## Discussion

In both patients, a second homozygous variant explained features beyond *COL4A6*-associated HL, highlighting the value of genome-wide testing in complex clinical presentations. In the proband of Family 1, a homozygous *HEXA* variant (NM_000520.6: c.902T>G p.(Met301Arg)), previously associated with both infantile and juvenile forms of Tay-Sachs disease, is likely to underlie the neurological decline, including vision loss, motor regression, and respiratory issues. This variant is located within the catalytic domain of the α-subunit of β-hexosaminidase A and has been hypothesized to impair subunit association, potentially disrupting enzymatic function. While its precise biochemical impact remains unclear, clinical reports describe affected individuals presenting with neurodegenerative symptoms such as gait ataxia, dysarthria, and bilateral tremors, often with disease onset around 5 years of age.^24^ These features are consistent with the proband we describe. Based on available data and ACMG/AMP criteria, the variant is considered likely pathogenic. In the proband of Family 2, a previously reported homozygous splice-site variant in *DYM* (NM_001353214.3: c.1125 + 1G>T p.?) was identified that was classified as likely pathogenic. Variants in *DYM* are associated with DMC disease, a rare skeletal dysplasia characterized by short stature, postnatal microcephaly, coarse facial features, psychomotor delay, and variable intellectual disability. These features were present in our proband and his affected brother. Literature reports of individuals with pathogenic *DYM* variants, including nonsense or splice-site changes, describe overlapping phenotypes such as a short trunk and limbs, pigeon chest, abnormal gait, joint contractures, dysarthria, apraxia of speech, intellectual disability, and skeletal anomalies like platyspondyly, lacy iliac wings, and epiphyseal irregularities.^25^ Although detailed radiographic evaluation was not available for affected individuals, the clinical presentation is consistent with DMC, further supporting the pathogenic role of the *DYM* variant identified. These findings underscore the diagnostic value of comprehensive genomic analyses, particularly in cases presenting with overlapping or multisystem features.

Interestingly, a detailed examination of *Col4a6* expression in wild-type and knockout mouse cochleae has shown that loss of COL4A6 does not result in significant cochlear structural abnormalities or severe HL.^3^ Auditory brainstem response thresholds were normal, and cochlear morphology appeared intact, indicating that the absence of the α6(IV) chain alone does not replicate the HL and cochlear malformation observed in humans with *COL4A6* variants. This suggests that human pathogenic variants likely act through dominant-negative effects rather than simple loss-of-function.^3^ Missense or splice variants may disrupt the formation or stability of the α5α6α5(IV) collagen heterotrimer. These abnormalities are likely to result in compromised basement membrane integrity within the inner ear, which can disrupt the structural support and signaling functions of the cochlear basement membranes ultimately impairing hair cell function and contributing to sensorineural HL.^26^ Our results are consistent with previously reported cases (Supplementary Table S2). In the originally described Hungarian family, the *COL4A6* c.1771G>A p.(Gly591Ser) variant led to prelingual, bilateral, severe sensorineural HL and cochlear malformations with incomplete separation of the cochlea in hemizygous males, while heterozygous females showed milder, variable HL in adulthood.^2^ Similar phenotypes were also reported in individuals with either a *COL4A6* c.3272G>C p.(Gly1107Ala) missense variant affecting Gly-X-Y repeats or a canonical splice variant c.951 + 1G>T causing in-frame exon skipping.^5^ Moreover, a patient with a c.1456G>A variant in *COL4A6* also reported cochlear hypoplasia and profound HL, further expanding the phenotypic spectrum.^27^

The Gly-X-Y repeat motif is central to the structural integrity of all type IV collagens. Its disruption often compromises triple-helix formation, resulting in misfolding or aggregation.^28^ In the context of α5α6α5(IV) networks, this can perturb basement membrane architecture in the inner ear, contributing to auditory dysfunction.^29^ This aligns with prior *COL4A6* reports (Supplementary Table S2), suggesting that the auditory phenotype may be more selectively impacted in certain variants or contexts.

Notably, the two *COL4A6* variants we identified (NM_033641.4: c.1480G>A p.(Gly494Arg) and (NM_033641.4: c.1767G>A p.(Pro589=)) are predicted to disrupt Gly-X-Y motifs through direct amino acid substitution or leaky splicing, respectively, potentially destabilizing the triple-helical structure of alpha-5 and alpha-6 chain isoforms that self-assemble into a triple-helical network. To further investigate the impact of the p.Gly494Arg substitution, we performed structure-guided modeling of the collagen IV α5α6α5 triple-helix. Introduction of the bulky, charged arginine residue at the invariant glycine position resulted in local structural distortion and steric clashes, consistent with destabilization of the helix.

Furthermore, we describe a patient with a synonymous *COL4A6* variant, highlighting an underappreciated variant class that can complicate genetic diagnoses. Although conventionally considered neutral, synonymous variants are known to affect gene expression through diverse mechanisms, including disruption of RNA splicing, alteration of mRNA secondary structure or stability, and changes in codon usage that impact translation efficiency.^30^ Emerging evidence suggests that 5–10% of human genes harbor functionally sensitive regions within their coding sequences where synonymous substitutions can disrupt regulatory elements such as exonic splicing enhancers or codon optimality, thereby contributing to disease risk.^31^ These findings challenge the long-standing assumption that synonymous substitutions are benign and highlight the importance of assessing potential pathogenicity. In our case, functional testing of the c.1767G>A (p.Pro589=) variant using a minigene assay demonstrated leaky splicing (r.1588_1767del p.Gly530_Pro589del), providing direct evidence of its impact on RNA processing and supporting its likely role in disease. The substituted residue located at the exon 22-intron junction is typically involved in base pairing with the 5′ end of the U1 snRNP to form the extended U1-5′ splice site duplex at the earliest stages of the splicing cycle.^32–34^ The duplex typically consists of 9 bp, of which, three involve exonic nucleotides (–3, –2, –1). The favored G-1 base would be flipped out from the U1-5′ splice site duplex in the c.1767G>A patient substitution, likely reducing the probability of the recruitment of the U1 snRNP to the weakened 5′ splice site (Figure 2E).^35^^,^^36^ It is plausible that the observed exon skipping could be explained through such a mechanism. Collectively, this reinforces the need for experimental validation of synonymous variants in clinical genomics, where their consequences might otherwise be overlooked.^37^

In Family 2, the mother (III:2) presented with mild HL (borderline normal) in her left ear, which may either represent variable expressivity in a heterozygous carrier or normal decline with age. Indeed, in X-linked HL genes, such as DFNX6 (*COL4A6*), female carriers display a wide spectrum of phenotypes, ranging from unaffected to moderate HL and with age of onset that may be delayed.^2^ Cases of X-linked Alport syndrome similarly demonstrate that ∼10% of female carriers develop HL by middle age, with significant variability in severity and progression.^38^ One plausible mechanistic explanation is skewed X-inactivation. For example, animal models of Alport syndrome show that preferential inactivation of the mutant allele correlates with milder disease,^39^ and skewed inactivation patterns have been implicated in variable expressivity in other X-linked disorders such as *SBMA* and *CMT1X.*^40^ Although X-inactivation patterns were not assessed, acknowledging this mechanism and reviewing relevant literature, these observations support a possible hypothesis role for skewing as a modifier of phenotypic severity in our case.

Dual molecular diagnoses present a unique challenge in clinical practice, as patients may have two distinct genetic disorders contributing to overlapping symptoms, complicating the diagnostic process.^41–43^ When the clinical picture is atypical or unexpected, the possibility of a dual diagnosis should be considered. As targeted gene panels may miss variants in genes not included in panel design, exome and genome sequencing could provide a broader genomic view to enable accurate identification of dual diagnoses and a better understanding of the patient’s condition, ultimately improving diagnosis, treatment strategies, and genetic counseling.^44^

While this study provides insights into the pathogenic role of new *COL4A6* variants, several limitations should be acknowledged. Firstly, the functional validation of the splicing effect was limited to an *in vitro* minigene assay. Although this approach demonstrated leaky splicing, it may not fully recapitulate endogenous splicing events in the inner ear tissues. Ideally, patient-derived RNA from relevant tissues would provide a more physiologically accurate assessment, but this was not feasible due to tissue inaccessibility. Furthermore, other detrimental impacts of the variant, such as abnormal RNA regulation, mRNA structure, and translation kinetics, are not directly measured from this assay. Secondly, although we performed homology modeling, the protein-level or functional consequences of the variants were not directly assessed. Experimental validation using patient-derived cochlear models or heterologous expression systems is needed to determine their impact on collagen IV assembly, stability, and potential dominant-negative effects. Thirdly, due to the small sample size, it is not possible to draw broader genotype–phenotype correlations. However, as neither *DYM* nor *HEXA* cause hearing impairment that is solely attributed to the *COL4A6* variants, our work contributes to the clinical and molecular picture of *COL4A6*-associated hearing impairment. Additionally, given the X-linked inheritance pattern, further investigation is necessary to elucidate the variable expressivity and penetrance, particularly in female carriers. Lastly, while our data support a likely pathogenic classification of the variants, further mechanistic studies, such as the use of animal models or inner ear organoids, are required to understand their impact on cochlear development and auditory function and to confirm or disprove a dominant-negative mechanism.

## Conclusions

This study presents two *COL4A6* variants associated with HL, each contributing to complex phenotypes through distinct molecular mechanisms. Our findings highlight the importance of comprehensive genetic evaluation, including functional RNA studies, especially for interpreting synonymous variants that may disrupt gene function by altering the splicing process. Additionally, we underscore the value of genome wide testing in uncovering dual diagnoses that explain complex phenotypes.

## Supplementary Material

hcaf246_Supplementary_Data
