# Supplementary material for: Uncovering dual molecular diagnoses in families with complex phenotypes through structural and clinical studies of novel COL4A6 variants
Source: QJM. 2025 Oct 15;119(3):187–97. doi: 10.1093/qjmed/hcaf246 (PMC13070642; doi:10.1093/qjmed/hcaf246)
Supplement: hcaf246_Supplementary_Data [file hcaf246_supplementary_data.zip › List of abbreviations.docx]

List of abbreviations

AAALAC Association for Assessment and Accreditation of Laboratory Animal Care

ACMG/AMP American College of Medical Genetics and Genomics/Association for Molecular Pathology

CADD Combined Annotation Dependent Depletion

dpf day post-fertilization

HGMD Human Gene Mutation Database

HL Hearing loss

hpf Hours post fertilization

IACUC Institutional Animal Care Committee

PTA Pure tone average

qRT-PCR Quantitative real time PCR

WISH Whole-mount in situ hybridization
