# Supplementary material for: Uncovering dual molecular diagnoses in families with complex phenotypes through structural and clinical studies of novel COL4A6 variants
Source: QJM. 2025 Oct 15;119(3):187–97. doi: 10.1093/qjmed/hcaf246 (PMC13070642; doi:10.1093/qjmed/hcaf246)
Supplement: hcaf246_Supplementary_Data [file hcaf246_supplementary_data.zip › Supplementary Tables_Final.docx]

**Supplementary Table 1.** Primers used for confirmatory and functional studies

| Region of Interest | Primer Name | Primer Sequence 5´- 3´ | Product Size |
| --- | --- | --- | --- |
| *COL4A6* Exons 21 | Hu COL4A6 Ex21 F | TTCCATTGCTTCCTCAGGGTCA | 317 bp |
|  | Hu COL4A6 Ex21 R | GCAAGCCTTCAGATTCCCTCTATGG |  |
| *COL4A6* Exons 22-23 | Hu COL4A6 Ex22 F | ATAGGGGTGAGGAGACAGGG | 774 bp |
|  | Hu COL4A6 Ex22 R | CAATGTGCTGGCCCATGAAG |  |
| *DYM* Exon 10 | Hu DYM Ex10 F | GCGAACTATCCCAAGGACAA | 485 bp |
|  | Hu DYM Ex10 R | CAGTCCTTTCCCCTCATCAA |  |
| pSPL3 exons A and B | SD6 F | TCTGAGTCACCTGGACAACC | 437 bp/  257 bp |
|  | SA2 R | ATCTCAGTGGTATTTGTGAGC |  |
| *COL4A6* Exons 22-23 | Hu COL4A6 Ex22-23 XhoI F | aattctcgagGTGCCAATATGTACCCAAGG | 471 bp |
|  | Hu COL4A6 Ex22-23 BamHI R | attggatccTTTCTGGATAGGGGTGAGGA |  |
| *COL4A6*-specific WISH primers | COL4A6-specific WISH F | gaattgaattaaccctcactaaagggCCACTTGGATTCAGCAACAG | 858 bp |
|  | COL4A6-specific WISH R | gaattgtaatacgactcactatagggCCCATCAAAGCCTTTAGCTCC |  |
| *COL4A6*-specific RT-qPCR primers | COL4A6-RT-qPCR F | GTTGGTCCACAAGGAGTCAGG | 255 bp |
|  | COL4A6-RT-qPCR R | GCCTGGATAACGAAATGGGTTTACT |  |

Transcripts used: *COL4A6*: NM_033641.4, *DYM*: NM_001353214.3. Abbreviations: WISH, whole mount in situ hybridization

**Supplementary Table 2.** Current and previously reported COL4A6 variants

|  | **Database/**  **tool** | **Family 1**  **Proband** | **Family 2**  **Proband** | **Rost et al.,**  **2014** | **O’Brien et al.,**  **2022** | **O’Brien et al.,**  **2022** | **Feng et al,**  **2024** |
| --- | --- | --- | --- | --- | --- | --- | --- |
| **Position**  **(GRCh37)**  **NM_001287758.2** | g. position | g.107431854C>T | g.108187848C>T | g.108187279C>T | g.108195081C>A | g.108171395C>G | g.107431878C>T |
|  | c. position | c.1480G>A | c.1767G>A | c.1768G>A | c.948+1G>T | c.3320G>C | c.1456G>A |
|  | p. position | p.(Gly494Arg) | p.(Pro589=) | p.(Gly590Ser) | p.(Pro303Glnfs*11) | p.(Gly1107Ala) | p.(Gly486Ser) |
|  | Exon | 21 | 22 | 23 | 15 | 34 | 21 |
| **Reference Databases** | dbSNP | rs1341885508 | rs781038243 | rs779748859 | rs1235090929 | [rs769241359](http://www.ncbi.nlm.nih.gov/snp/rs769241359) | rs769966341 |
| **Population frequency databases** | gnomAD v4.1 | Female: 0.000002473  Male:  0.000007758  Overall MAF: 0.000004182 | Female: 0.00002570  Male:  0.00002985 Overall MAF: 0.00002695 | Female: 0.00003857  Male: 0  Overall MAF: 0.00002684 | Female: 0.000008689  Male: 0  Overall MAF: 0.000005638 | Female: 0.0002338  Male:  0.0001367  Overall MAF: 0.0001985 | Female: 0.000002493  Male:  0.000005345 Overall MAF: 0.000003400 |
| **Pathogenicity prediction tools** | CADD Phred | 23.4 | No result | 31 | No result | 23.2 | 23.3 |
|  | Mutation  Taster | Benign | No result | Benign | No result | Benign | Benign |
| **Splicing Predictions** | Total | 0.0% | -11.1% | -17.2% | -100.0% | 0.0% | 22.4 |
|  | MaxEntScan | 0.0% | -16.7% | -27.8% | -100.0% | 0.0% | Benign |
|  | NNSPLICE | 0.0% | -3.3% | -19.1% | -100.0% | 0.0% | 0.0% |
|  | SSF | 0.0% | -13.3% | -4.9% | -100.0% | 0.0% | 0.0% |

Note: a possible discrepancy in the c. and p. position of previously published variants is due to different transcripts that were used for reporting. All variants were annotated using the transcript NM_001287758.2 that is also used in the Deafness Variation Database v9.
