# Supplementary figures and images for: Uncovering dual molecular diagnoses in families with complex phenotypes through structural and clinical studies of novel COL4A6 variants
Source: QJM. 2025 Oct 15;119(3):187–97. doi: 10.1093/qjmed/hcaf246 (PMC13070642; doi:10.1093/qjmed/hcaf246)

*col4a6* mRNA expression

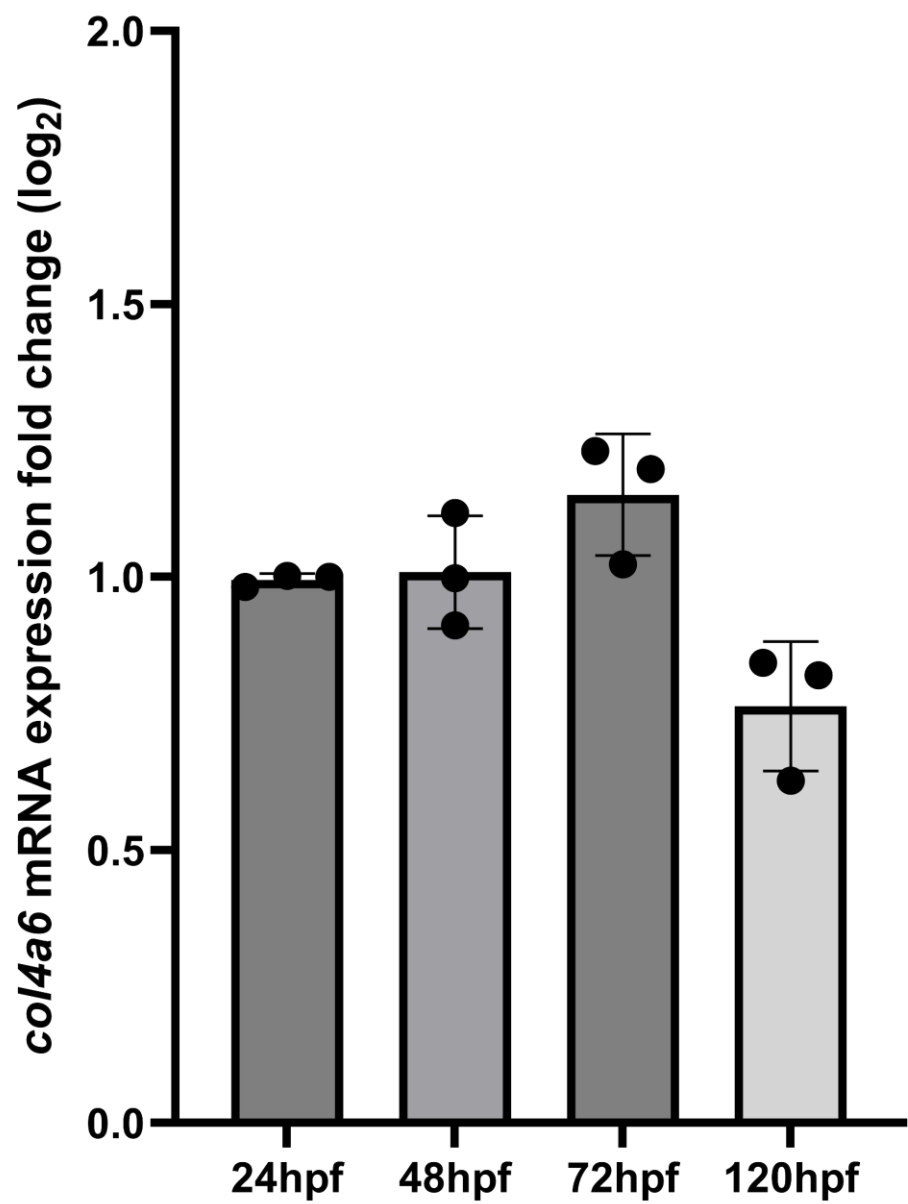

Supplement: hcaf246_Supplementary_Data [file hcaf246_supplementary_data.zip › Supplementary Figure 1.pdf]
